# Feelings of Not Mattering and Moral Injury Distress in University Students Exposed to Traumatic Events and Adverse Childhood Experiences

**DOI:** 10.3390/ijerph23070917

**Published:** 2026-07-17

**Authors:** Heather Lumsden-Ruegg, Anthony M. Battaglia, Gordon L. Flett, Stephan Bonfield, Joel O. Goldberg

**Affiliations:** Department of Psychology, York University, Toronto, ON M3J 1P3, Canada; hruegg@yorku.ca (H.L.-R.); abattag@yorku.ca (A.M.B.); gflett@yorku.ca (G.L.F.); spbonfie@ucalgary.ca (S.B.)

**Keywords:** university students, 2SLGBTQIA+, moral injury emotions, anti-mattering, mattering, psychosocial trauma

## Abstract

**Highlights:**

**Public health relevance—How does this work relate to a public health issue?**

- Trauma exposure and Moral Injury (MI) distress are widespread issues that affect mental health outcomes at a population level.
- Feelings of mattering versus not mattering are closely related to psychological well-being and can be seen as social determinants of health, particularly for vulnerable groups like 2SLGBTQIA+ students.

**Public health significance—Why is this work of significance to public health?**

- The study informs potential community prevention and clinical intervention strategies by identifying key risk and protective factors that influence mental health, such as adverse childhood experiences, anti-mattering, and under-recognized MI emotional distress.
- Disparities in trauma exposure and distress are identified among 2SLGBTQIA+ university students, emphasizing the demand for equity-focused mental health supports that address the anti-mattering findings that their voices feel unheard.

**Public health implications—What are the key implications or messages for practitioners, policy makers and/or researchers in public health?**

- Interventions should prioritize proactive reaching out, fostering a sense of community, of mattering, of allowing voices truly “being heard” to reduce the distress associated with feeling insignificant in order to improve mental health outcomes in this trauma-exposed student population.
- Policies and programs should be tailored to address the heightened risks faced by 2SLGBTQIA+ individuals in order to promote inclusive, trauma-informed, and culturally sensitive care, incorporating sensitivity to MI-informed emotional complications.

**Abstract:**

Many university students have a history of exposure to adverse and potentially traumatic life events and may therefore experience Moral Injury (MI). University students exposed to trauma, and especially relational trauma, should be less impacted if they feel and know with certainty that they are valued and matter to other people. The current study uniquely examined MI distress and its correlates in 382 university students who reported the experience of at least one potentially traumatic event. Additionally, we examined the traumatic experiences faced by a subgroup of 2SLGBTQIA+ students and their associated reactions. Various self-report measures were administered including MI emotions, mattering, anti-mattering, and adverse childhood experiences (ACEs). Our analyses confirmed that MI distress was evident and was linked with ACEs and higher levels of anti-mattering. Main findings accord with the notion that traumatic interpersonal events are not only distressing but are connected with feeling unimportant and insignificant to others. In addition, comparisons with the sexual and gender non-minority subgroups established that 2SLGBTQIA+ students were exposed to higher rates of major life stressors and they had comparatively elevated feelings of not mattering to others and MI-related emotional distress. Collectively, the results underscore the heterogeneity that exists among students and that feelings of mattering versus feelings of not mattering can be a source of strength or vulnerability. Clinical and public health implications are discussed as relevant to addressing feelings of personal insignificance and moral-injury related shame and mistrust.

## 1. Introduction

It is important to acknowledge the heterogeneity that exists when considering the mental health and well-being of college and university students. Most students are emerging adults who face various forms of stress due to acute and chronic stressors [1] and daily stressors and hassles [2] along with the general stress that accompanies the transition to university [3]. Many students also experience chronic pressure [4] and stress that is self-generated [5]. Unfortunately, some students have also experienced traumatic events, with a history of abuse or assault being far too common. Some estimates suggest that as many as one in five students experience events that involve clinical or sub-clinical post-traumatic stress [6].

The current study was designed to illuminate the variation in student well-being by focusing on mattering and its links to both exposure to potentially traumatic events and the resulting sense of Moral Injury (MI). Mattering is the feeling of being valued by other people in ways that leave one feeling seen, heard, and appreciated [7,8]. Mattering is double-edged in that it is highly protective, but fears and feelings of not mattering can be very painful [9]. Mattering may serve as a protective resource in roles and contexts where individuals are exposed to potentially traumatic experiences that can evoke distress and MI. MI is typically studied in high-risk occupational contexts such as the military, public safety, and healthcare [10]. What is MI? MI occurs when one’s values are violated, typically following perpetrations (e.g., when an individual personally acts, witnesses an act, or fails to act in a way that counters their moral code) or betrayals (e.g., when an individual in a position of authority fails to provide protection or defense in a time of need or crisis) [11,12]. MI is underpinned by guilt, shame, anger, loss of trust, self-criticalness and low self-worth, negative beliefs about others, and spiritual/existential crises [13,14], and has been linked to PTSD, depression, anxiety, substance use, and suicidality [10,15].

At present, there is a growing recognition of the relevance of MI within civilian populations, and recent advances have been made in its study among civilian [16] and medical student samples [17]. However, little is known about how MI is experienced in university students and emerging adult populations. Young adults can have traumatic life experiences and may at times feel as though their moral code has been violated through their own actions or the actions of others, resulting in feelings of shame, guilt, and blame. One would expect that this phenomenon would be especially evident among young people who have the misfortune of experiencing abuse and neglect in their childhoods or recent past or who have been subjected to some form of assault. We sensed that by studying MI emotions among university students, we would gain a better understanding of those students who are outwardly managing but are internally struggling as a result of their traumatic life experiences and their negative feelings about the world and themselves.

The current research had three main goals. First, and foremost, we sought to establish that it is valid and meaningful to consider MI distress in students while also getting some sense of the extent to which they have experienced traumatic events. This seems essential given the lack of focus on MI in students thus far in the literature. Second, we considered some potential key correlates of MI distress with a psychosocial basis—adverse childhood experiences and feelings of mattering versus not mattering to other people. A third goal due to its anticipated relevance was to compare and contrast the experiences and well-being of students who might be especially vulnerable. Specifically, as discussed in more detail below, we consider students who identify among people representing sexual and gender minorities.

### 1.1. Adverse Childhood Experiences

Our focus on adverse childhood experiences (ACEs) is a reflection of extensive evidence that links a history of ACEs and vulnerability to moral injuries in various populations [18,19,20]. How does the prior experience of ACEs contribute to a vulnerability to MI and associated distress reactions? One plausible explanation is that continued exposure to ACEs can shape the young person’s sense of identity as being someone who is weak and bad, rendering one incapable of being able to proactively address and resolve factors that contribute to trauma and negative experiences [21]. Perhaps the MI distress response is also amplified when current adverse experiences trigger rumination and upsetting autobiographical memories. Given these observations, the inclusion of a measure of ACEs in the current study seems highly justified.

Below, mattering is described in more detail. We then outline its potential relevance in the current work and specific predictions tested in the current study.

### 1.2. Mattering

Mattering is the feeling of being significant to other people. Rosenberg and McCullough [8] introduced the construct and posited that everyone has a need to feel important to others. They noted that it is distressing to be treated as unimportant by people, especially when this treatment comes from people who are important to us. Initial research established that mattering to parents is linked negatively with distress in adolescents [8] and mattering predicts reduced vulnerability to depression in longitudinal research with adults [22].

While mattering has typically been studied as a positive psychology concept and has been linked with happiness and well-being [23], recent advances have added a focus on anti-mattering [7,9] which is the tendency to feel invisible, irrelevant, and unimportant to others. Anti-mattering is recognized as more than the opposite of mattering, with various distinguishing factors, including different motivational orientations. Research has established broadly that low levels of mattering and high levels of anti-mattering are associated with various forms of psychological distress and anti-mattering often predicts significant variance in key outcomes beyond mattering [24,25,26,27,28].

Why should feelings of anti-mattering be linked with MI and psychological distress? Some research suggests that MI is likely when core psychological needs, including the need for connection, are not met [29]. In theory, this should extend to violations of the need to matter to others. The MI experience and events leading up to it are not in keeping with key needs that people both hope for and expect to be satisfied. The significance of mattering in university students is well-established [28,30]. A related possibility relevant to the feeling of not mattering is that the pain of MI and failing to do something to avert or terminate traumatic experiences can evoke feelings of shame for being insignificant and ineffective in the eyes of other people as well as oneself. For example, one might think, “A good person is depended on by others, and I am not someone who can be relied on.” It has been proposed that the cognition of not being cared about by other people is tied closely to the emotional reactions of people who have experienced MI [31]. These possibilities combine to suggest that feelings of not mattering should be linked with MI distress and pain. Despite this hypothesized connection, to our knowledge, no published research has examined how needing to be significant and to matter to others relates to the presence or absence of MI in university students.

Parenthetically, while it is not the main focus, there is little published research on the antecedents of feelings of mattering versus not mattering, and the potential role of adverse childhood experiences has not been investigated. However, some research with emerging adults has linked a reported history of child maltreatment involving abuse and neglect with reported levels of mattering [32]. Accordingly, it is possible in the current research that anti-mattering and ACEs will be positively associated.

### 1.3. The Relevance of Focusing on the 2SLGBTQIA+ Community

Individuals in the Two-spirit, Lesbian, Gay, Bisexual, Transgender, Queer, Intersex, Asexual, and other identities (2SLGBTQIA+) community face unique life stressors and adversity beyond what is experienced by their cisgender and heterosexual counterparts [33]. The acronym has evolved over time to become more inclusive, reflecting broader recognition of diverse sexual orientations and gender identities. We recognize that Two-Spirit identities are grounded in Indigenous cultural understandings of gender and sexuality and may involve experiences distinct from those captured by Eurocentric gender and sexual identity frameworks. In the following review of the literature, we retain the specific terminology and acronyms used by authors in their respective studies. The minority stress model proposed by Meyer [34] aims to recognize these major sources of stress within sexual minority populations. Sources of minority stress can be objective (e.g., prejudicial events, including harassment or assault) or subjective (e.g., stigma resulting in vigilance when expecting rejection or discrimination). The model offers some explanation for the increased levels of psychological distress routinely observed in these populations. For example, it has been estimated that gender minority young adults are 4.3 times more likely and sexual minority individuals are 2.2 times more likely to meet criteria for a mental health disorder than those who identify as cisgender and heterosexual [35,36]. Sexual minority individuals who have experienced prejudicial forms of minority stress also have an increased likelihood of poor physical health [37]. Though Meyer’s [34] model was developed with sexual minority individuals in mind, more recent examinations of minority stress extend the model to gender minority individuals as well. For instance, minority stress in LGBTQ youth has been linked to an increased risk for symptoms of post-traumatic stress disorder (PTSD), depression, and suicidality [38]. In the current study, we examined life stress among sexual and gender minority university students and how these experiences may relate to indicators of psychosocial distress.

Individuals in the LGBTQ+ community are also known to experience high rates of adverse childhood experiences (ACEs), with an estimated 86% experiencing at least one and 43% experiencing more than four potentially traumatic events in childhood [39]. Since longitudinal studies have linked ACEs with higher rates of depressive symptoms, drug use, and antisocial behaviour in early adulthood [40], it is critical to consider how this may impact vulnerable groups. Additionally, sexual or gender minority children may experience ACEs that are specific to their marginalized status, and this has been linked to lower perceived social support in later life [41]. Of concern, reduced social support has been connected to increased suicide risk and self-harm in sexual and gender minority populations [42]. Given the negative outcomes associated with minority stress and ACEs, it is important to develop a stronger understanding of the experience of trauma within the 2SLGBTQIA+ community.

Individuals in the 2SLGBTQIA+ community are acutely at risk for potentially morally injurious events. They may experience internal turmoil where their values conflict with those of their family or the broader societies that they interact with. One example that draws attention to the possibility of MI in this population is the self-stigmatization involved in internalized homophobia, where an individual assimilates broader social values to the detriment of their sense of self [34]. They may feel betrayed (e.g., having love and affection withheld by family members) or as though they have personally committed acts that conflict with their moral code (e.g., choosing to hide their sexual or gender minority identity from peers). These experiences could result in emotional distress in the form of shame or guilt, which are distinct features of MI known as MI emotional sequelae or MI emotions. Notably, the experience of shame and guilt has been found to act as a barrier to care-seeking among populations in which MI has been investigated [15].

In terms of relevant literature, a recent mixed-methods study by Nicholson and colleagues [43] examined dimensions of MI and minority stress in sexual and gender minority adults. The qualitative analysis identified themes related to MI including shame, guilt, betrayal and loss of trust, along with the unique feature of “attachment injuries” that potentially contribute to the experience of MI in these individuals. Shame has also been explored in the 2SLGBTQIA+ community outside of the MI perspective. For example, one study examined LGBTQ community members who endorsed at least one potentially traumatic life experience and found that shame partially mediated the relationship between exposure to traumatic events and physical and mental health [44]. Individuals who reported higher levels of shame also reported more severe symptoms of depression, PTSD, substance use, chronic health conditions, somatic symptoms, and risky sexual behaviours.

One further possibility tested uniquely in the current study is that feelings of not mattering to others will be elevated among students in the 2SLGBTQIA+ community, and these feelings of not mattering will be linked with emotional reactions to MI among our participants. Mattering has been recognized as a source of resilience and is valued as a protective agent against negative mental health outcomes. For example, in older gay men, mattering has been found to partially mediate the relationship between internalized gay ageism and symptoms of depression [45]. Additionally, higher mattering has been associated with higher social and psychosocial well-being among sexual and gender minority adults [46]. Mattering occurs and can be measured across multiple contexts, including school or work. Pousson and O’Laughlin [47] conducted a narrative inquiry with a small group of LGBTQIA community college students to investigate the influence of marginality and mattering in their academic environment. Sexual and gender minority students noted mixed feelings about their sense of mattering with faculty, with some instructors acknowledging and supporting their minority status and others failing to do so. These students sensed a general absence of recognition from the administration, with issues such as a lack of gender-neutral bathrooms and an inability to update their preferred names in the college’s system. Despite these negative findings, post-secondary settings may also provide unique opportunities for building resilience through mattering. Sexual minority university students report that school settings helped them to assert their identities, gain support and a voice through campus organizations, and find opportunities to make meaningful connections with similar others [33].

Anti-mattering concerns, such as feeling invisible to others and having voices unheard, should be particularly relevant among our participants. Among LGBTQ+ populations, anti-mattering has been explored as a potential correlate to suicidal ideation. Using a grounded theory approach, Deas and colleagues [48] investigated an online peer-support forum for individuals contemplating suicide. They found that over 93% of the posts left by individuals who identified as part of an LGBTQ+ group included what could be considered anti-mattering content, with statements such as “Even if you don’t really mean it, could someone tell me that I matter?” Additionally, 68% of these posts mentioned the MI emotion of shame. Taken together, these findings are suggestive of the possibility that anti-mattering may be related to MI emotions, though this possibility has not yet been explored explicitly.

### 1.4. The Current Study

To summarize, the current study had the main goals of examining MI distress and possible mattering and anti-mattering correlates in university students who disclosed trauma exposure. While our overarching focus was on the entire sample of students, we also sought to explore how a 2SLGBTQIA+ sub-group population might experience MI emotions alongside mattering and anti-mattering.

## 2. Materials and Methods

This online study followed a cross-sectional, observational design and was conducted as part of a larger body of research investigating MI and other factors related to well-being. Ethics approval was obtained through the York University Human Participants Research Committee (REB Certificate #: 2022-350), and participants provided their written informed consent online before participating in the study. Data collection occurred between 3 March and 11 April 2023.

### 2.1. Participants & Procedure

A total of 651 undergraduate students at a large Canadian university participated in the current study. Participants were recruited through an undergraduate research participant pool, through which students enrolled in certain undergraduate courses can choose from a variety of approved studies and receive course credit for participation. Responses were screened and participants were excluded for failing to answer more than two attention check questions correctly (*n* = 95), responding too quickly (<8.3 min) or taking too long to respond (>24 h) (*n* = 28), or reporting that they did not pay sufficient attention for their responses to be valid (*n* = 65). A total of 463 valid profiles were identified.

Participants were further screened for lifetime trauma exposure, with 382 (82.5% of valid profiles) students reporting exposure to at least one potentially traumatic event in their lifetime. Most participants were women (*n* = 287; 75%), not members of the 2SLGBTQIA+ community (*n* = 316; 83%), and indicated they were single (*n* = 346; 90%). Notably, all participants who reported being a member of a gender minority group also reported belonging to the 2SLGBTQIA+ community. Fifty-five percent (*n* = 204) of participants self-identified as members of a racialized group. Response options were “yes”, “no”, or “prefer not to answer”, and nine percent (*n* = 34) preferred not to disclose whether they identified as a member of a racialized group. A numeric treatment was applied to our categorical age variable, revealing an approximate mean age of 22.3 years (*SD* = 3.6). Sociodemographic information in the sample is displayed in Table 1.

All data were gathered anonymously through an online self-report survey using Qualtrics. After providing informed consent, participants provided some basic socio-demographic information and completed a battery of survey measures assessing known and putative mental health risk and resilience factors, described in detail below. The Conscientious Responders Scale (CRS) is a measure designed to identify potentially inattentive or careless responding in survey research (e.g., “To respond to this question, please choose option number five, ‘slightly agree’”) [49,50]. Items from the CRS were interspersed throughout to measure attentive responding. The entire survey was estimated to take 30 min to complete. Upon completion of the survey, contact information for mental health resources was provided in case participants experienced increased psychological distress and needed support. Participants received course credit for participation.

### 2.2. Measures

#### 2.2.1. Exposure to Traumatic Events

The Life Events Checklist for the Diagnostic and Statistical Manual-5 (LEC-5; [50]) was used to assess the degree or extent of exposure to major life stressors and potentially traumatic events in a respondent’s lifetime. This 17-item checklist instructs respondents to select whether they have ever encountered events involving various types of assault (i.e., physical assault, sexual assault, assault with a weapon), other discrete events (e.g., fire, traffic accident, serious accident at home or work), traumatizing natural disaster, “severe human suffering” or “captivity.” Other events include witnessing a sudden violent death or sudden accidental death. Because our study sought to examine outcomes related to well-being in students who had prior exposure to potentially traumatic experiences, only participants who endorsed at least one item on the LEC-5 were included in the study. A continuous variable comprising total events reflecting the sum of endorsed items was used to reflect instances of possible exposure to trauma.

#### 2.2.2. Adverse Childhood Experiences

The Adverse Childhood Experiences Questionnaire (ACE; [51]) assessed exposure to negative events in the first 18 years of life. We administered an expanded 10-item version of this scale similar to Merrick and colleagues [52], assessing neglect, abuse, and household dysfunction; however, the item assessing spanking was not included. Participants were asked to respond “Yes” or “No” to questions such as “Did a parent or other adult in the household often or very often push, grab, slap, or throw something at you? Or ever hit you so hard that you had marks or were injured?” A sum score (yes = 1; no = 0) with a possible range of 0 to 10 was used to gauge the extent to which participants experienced potentially traumatic events in their early life.

#### 2.2.3. Moral Injury (MI)

MI emotions were examined using the emotional sequelae subscale of the Moral Injury Assessment for Public Safety Personnel (MIA-PSP; [20]). The subscale consists of 7 items measuring emotional distress resulting from exposure to potentially morally injurious events (e.g., “I am bothered by feelings of shame”). Responses range from 1 = strongly disagree to 6 = strongly agree on a 6-point Likert scale. Sum scores are used to interpret the MIA-PSP. Higher scores represent higher levels of moral distress. The emotional sequelae subscale has possible scores ranging between 7 and 42. The original MIA-PSP development and validation found strong internal consistency for the overall scale (α = 0.93) and in its emotional sequelae subscale (α = 0.86; [20]). The current study found an internal consistency reliability of α = 0.92 for the subscale.

#### 2.2.4. Mattering

Mattering was measured using the General Mattering Scale (GMS; [53]). This five-item scale evaluates the extent to which someone feels they matter to others (e.g., “How important do you feel you are to other people?”). Participants respond using a 4-point Likert-type scale with responses ranging from 1 = not at all to 4 = a lot. Level of mattering is assessed through a sum score with values ranging between 5 and 20. Higher scores on this scale indicate that a respondent feels heard, appreciated and important to others. The GMS had an internal consistency reliability of α = 0.83 in the current study, which is consistent with previous work [7].

#### 2.2.5. Anti-Mattering

The five-item Anti-Mattering Scale (AMS; [9]) was used to measure the degree to which individuals feel they do not matter to others. For example, one item reads, “To what extent have you been made to feel like you are invisible?” The scale consists of five items and has respondents rate each item on a 4-point Likert-type scale (1 = not at all; 4 = a lot). A sum score is used to evaluate the level of anti-mattering, with possible scores ranging between 5 and 20. Higher scores indicate that a participant feels they are unimportant, underappreciated, and invisible to others. Since its development, the scale has been used globally and within assorted populations including students [54] and adults of various ages [26,55]. Initial validation of the AMS found an internal consistency reliability of α = 0.91, and the present study achieved α = 0.89.

### 2.3. Statistical Analysis

Statistical analyses were conducted using R Studio version 4.6.0 [56].The sample of *n* = 382 participants who endorsed at least one traumatic life event on the LEC-5 was broken down into two groups based on self-reported sexual and/or gender minority identity. This differentiation formed the basis for all later analyses. Participants who self-identified as having a sexual or gender minority status were designated the “2SLGBTQIA+” group, whereas those who did not were assigned to the “non-2SLGBTQIA+” group. Participants who selected “prefer not to answer” when reporting their sexual and gender minority status (*n* = 14) were excluded from the between-group analyses. Welch’s *t*-tests were then used to evaluate between-group differences in the MI emotional sequelae subscale, the ACE, the AMS, and the GMS. Additionally, between-group differences were examined for the demographic variables of gender, age, marital status, and racialized group status. A linear regression model was used to examine if MI emotions were uniquely determined by any of the other measures of interest. For all tests, α was set at 0.05. Effect sizes for group differences were calculated using Hedge’s g (due to unequal sample sizes) with cutoffs of 0.20, 0.50, and 0.80 representing small, medium, and large effect sizes, respectively [57]. Pearson’s correlation (*r*) was used to assess relationships between study variables with effect sizes designated as small (0.1), medium (0.3), and large (0.5) based on Cohen [58].

## 3. Results

Correlations between the ACE, the AMS, the GMS, and the MIA-PSP-ES for the final participants of interest, those endorsing trauma exposure, are reported in Table 2. Statistical significance was evaluated using a threshold of *p* < 0.05.

It can be seen that when focused on the emotional sequelae of MI, higher levels of distress were associated significantly with the reported experience of ACEs and anti-mattering, with medium effect sizes. There was also a significant link with the GMS, suggesting that greater mattering was associated with lessened MI emotional sequelae, though this correlation was small. Traumatic life events exposure was positively associated with ACEs to a medium degree, and with MI distress and anti-mattering to a small degree. Also, as postulated, there was a significant positive association with a medium effect size between anti-mattering and ACEs and there was a small effect size and negative association between ACEs and GMS scores. Correlations for each subgroup are reported in Table 3 and Table 4. The pattern of intercorrelations across the two groups was quite comparable, though it must be underscored that the correlations for the 2SLGBTQIA+ students was based on a smaller group of participants (*n* = 52).

### 3.1. Between-Group Comparisons

Participants in the non-2SLGBTQIA+ group had a mean age of 22.4 (3.83) years. Within this group, 9.5% indicated that they were married or cohabiting with a partner. Those in the 2SLGBTQIA+ group had a mean age of 21.9 (2.44) years, and similarly, 9.8% indicated that they were married or cohabiting. Age, *t*(97.95) = 1.09, *p* = 0.278, *d* = −0.13, 95% CI [−0.36, 1.23], and marital status, *X*^2^(1, *N* = 368) = 0.005, *p* = 0.94, were not significantly different between the two groups. The non-2SLGBTQIA+ group contained significantly more men than the 2SLGBTQIA+ group, *X*^2^(6, *N* = 368) = 72.48, *p* < 0.001, with no significant group differences in proportions of women. There were no significant differences in the proportion of participants who self-reported racialized group status, *X*^2^(2, *N* = 368) = 0.019, *p* = 0.99. Following this two-group design, means and standard deviations for the measures of interest in the current study are displayed in Table 5.

Based on total reported items on the LEC-5, participants in the 2SLGBTQIA+ group experienced a significantly higher number of traumatic life events compared with those in the non-2SLGBTQIA+ group, *t*(81.39) = −2.90, *p* = 0.005, *g* = −0.36, 95% CI [−2.26, −4.23]. Additionally, on average, those in the 2SLGBTQIA+ group reported significantly more adverse childhood experiences on the ACE, *t*(69.27) = −3.96, *p* < 0.001, *g* = −0.59, 95% CI [−1.95, −0.64]. Hedges’ *g* effect sizes were small for the LEC-5 and moderate for the ACE.

Group differences were also significant on the MI emotional sequelae subscale, with the 2SLGBTQIA+ group reporting higher levels of MI emotions than those in the non-2SLGBTQIA+ group, *t*(77.65) = −4.02, *p* < 0.001, *g* = −0.52, 95% CI [−9.58, −3.23]. A moderate effect size for MI emotions was observed. AMS score differences were statistically significant for the two groups as well, with higher levels of anti-mattering reported by individuals in the 2SLGBTQIA+ group, *t*(73.30) = −3.83, *p* < 0.001, *g* = −0.53, 95% CI [−3.22, −1.02]. The effect size for anti-mattering was moderate. In contrast, there was no statistically significant difference for the two groups on the GMS, *t*(64.09) = 0.60, *p* = 0.548, *g* = 0.10, 95% CI [−0.76, 1.41].

### 3.2. Regression Analyses

Multiple linear regression assessed whether anti-mattering, mattering, and ACEs were significantly associated with MI emotions for the 2SLGBTQIA+ group. We ran diagnostics to determine the best model fit, including added variable plots, component residual plots, and qq plots. The results of our best model fit are shown in Table 6. Holding GMS scores constant, AMS scores were significantly associated with heightened MI emotions within the 2SLGBTQIA+ group. Similar regression analyses conducted among non-2SLGBTQIA+ participants yielded a comparable pattern of significant associations; however, the magnitude of the association with anti-mattering was smaller than that observed among 2SLGBTQIA+ participants.

## 4. Discussion

The current study addressed multiple goals relevant to exploring protective and vulnerability factors in university student distress. First and foremost, we evaluated mattering, anti-mattering, and other predictors of emotional distress related to MI, while also attempting to illuminate the reality that many emerging adults who attend university have experienced one or more traumatic events. One overarching and novel result of the current research is that it both qualifies and uniquely extends the broader field of research on MI by uncovering the presence of negative emotions underpinning MI involving shame, guilt and distress in vulnerable university students. In this regard, we ascertained that a substantial proportion of students from our overall sample (*n* = 383) had experienced one or more traumatic events as assessed by the LEC-5. It was illuminating to determine that when we focused on those participants with at least one event, the overall mean for this subsample was remarkable at approximately six categories of potentially traumatic events.

Our results highlight the public health importance of assessing both current and past trauma among students presenting with symptoms of distress, as doing so may facilitate a better understanding of their exposure to potentially traumatizing experiences. The extent of possible trauma found among our participants is alarming, with 82.5% of valid profiles reporting at least one potentially traumatic life event. Clearly, university students are far from immune in terms of the potential experience of trauma, and this further points to the need to consider MI emotions in emerging adults.

Our correlational analyses that focused on our sample of university students exposed to traumatic life events suggest that those individuals with higher rates of ACEs felt that they did not matter as much to the people around them. We found uniquely in the current study that ACEs were linked significantly with anti-mattering. Moreover, the vulnerability factor of anti-mattering was associated significantly with MI emotions, while there was little relationship between mattering (which is considered a possible resilience factor) and either ACEs or MI emotions. The current results suggest that university students with elevated levels of anti-mattering are more prone to MI emotions such as guilt or shame. Such MI emotion findings have been under-recognized for these vulnerable distressed university students who are survivors of past trauma and who feel like they are insignificant to others and whose voices are unheard. As discussed below, trauma-informed interventions need to consider the shame and blame as possible MI distress and further, to be mindful of the moral-injury related mistrust arising in survivors from past hurts or betrayals.

Interestingly, the relationships between ACEs and anti-mattering and between ACEs and MI emotions were not as strong within the sexual and gender minority subsample when compared to the non-2SLGBTQIA+ group, with weak versus moderate correlations emerging, respectively. It is unclear, however, if this finding is an artifact of the small size of our 2SLGBTQIA+ group, or if there is an unknown factor contributing to the strength of this relationship. For both groups, there were moderate correlations between the AMS and the MIA-PSP-ES, indicating that individuals who reported feeling shame and guilt also tended towards feeling insignificant and invisible to the people in their lives. To the best of our knowledge, this study is the first to examine these variables together and among university students, therefore informing our understanding of the potential impact of ACEs on psychosocial functioning.

In addition to the correlations observed, we found that participants who identified as a member of a sexual or gender minority group reported more items on the LEC-5 and the ACE. This finding suggests that university students in the 2SLGBTQIA+ community are exposed to more traumatic events throughout their young lives than their non-2SLGBTQIA+ peers. Additionally, they reported significantly higher levels of anti-mattering and MI emotions, indicating they are experiencing distress in the form of shame, guilt, and feeling unimportant to others. The finding of increased ACEs and items on the LEC-5 aligns with previous literature, which has shown that 2SLGBTQIA+ individuals are at an increased risk of routine exposure to unique life stressors [33,34,38] and of experiencing potentially traumatic events, including experiences of family rejection, emotional abuse, and victimization [39,41]. Our results were consistent with past findings in that those in the 2SLGBTQIA+ group experienced an average of 1.3 more ACEs and 1.3 more items on the LEC than the non-2SLGBTQIA+ comparison group. This finding is notable, given the heightened risk for trauma-related outcomes that sexual and gender minority individuals may face. These disparities are often understood within minority stress and social determinants of health frameworks, which highlight how discrimination, marginalization, and unsupportive social environments can contribute to increased exposure to adverse experiences across the life course. Collectively the results of the current study further enhance our understanding of ACEs, mattering, anti-mattering, and MI emotions among trauma-exposed 2SLGBTQIA+ university students.

Consistent with our predictions, participants in the 2SLGBTQIA+ group reported experiencing significantly higher levels of MI emotions than their non-2SLGBTQIA+ counterparts. To our knowledge, this is the first study to look at MI emotions in trauma-exposed undergraduate students and within the context of gender and sexual minority status. In this context, MI emotions of shame and guilt may stem from potentially morally injurious events such as societal and familial pressures where an individual faces an expectation to “be” a certain way. Having an identity that conflicts with stigmatizing societal norms may lead an individual to feel as though they have done bad things (unfair self-blame) or that they are a bad person (shame), particularly if they feel their true self is unacceptable to the people around them. In line with this concept, authenticity around one’s sexuality among sexual minority populations has been linked to higher well-being along with decreased levels of stress and depressive symptoms, whereas concealment is related to negative mental health outcomes [59]. Our observation of higher MI emotions in the 2SLGBTQIA+ group is not surprising, given the complex social pressures known to impact gender and sexual minority individuals, as described in the minority stress model [34,38]. However, additional research will be needed to determine the source of heightened emotional distress within this population.

As suggested above, the current study found uniquely that trauma-exposed 2SLGBTQIA+ individuals experience comparatively higher levels of anti-mattering. The mean score of 14.2 found among the 2SLGBTQIA+ individuals far exceeds typical means and norms [7,9] and indicates that most respondents agreed to some degree with all five AMS items. Although such interpretation is based upon observations about descriptive statistics, this finding is especially disconcerting given the consistently lower levels found in previous research, established in broad samples of emerging adults, general adults, and adolescents that anti-mattering is linked with outcomes such as loneliness, depression, and suicidal ideation [9,27,55]. There is significant pain inherent in being made to feel irrelevant, insignificant, and invisible and it is vital to address the distress among these students. While feelings of not mattering are subjective and based on perceived importance or unimportance to other people, it is likely that for many 2SLGBTQIA+ individuals, the current results reflect veridical anti-mattering experiences.

Parenthetically, participants’ sense of mattering as assessed by the General Mattering Scale was not significantly different between groups. While not expected, this finding and other findings in the current study are in keeping with the contention that anti-mattering is not simply the opposite of mattering. While counter to our expectation that mattering would be higher for the non-2SLGBTQIA+ group, this null finding may be explained by the qualitative literature outlining social support as a source of strength and resilience within sexual and gender minority communities [33,47]. We tentatively suggest that participants in our study may have been well connected with similar others through campus organizations or elsewhere, leading to comparable levels of mattering between groups. In contrast, the level of anti-mattering was significantly different between groups, such that those in the 2SLGBTQIA+ group felt as though they mattered less to the people around them compared to those in the non-2SLGBTQIA+ group. This finding highlights the distinct characteristics of mattering and anti-mattering, in that one can feel important in some settings while simultaneously feeling invisible in others [9]. This observed elevated anti-mattering may reflect “dual identities” that gender and sexual minority individuals take on, where they are required to hide who they are in some contexts while at other times being able to present as their authentic selves. The known links between anti-mattering and negative mental health, including depression and suicidal ideation, make it important to identify the source of increased levels of anti-mattering felt among gender and sexual minority individuals.

Our regression analysis highlights anti-mattering as a unique and significant predictor of MI emotions for the 2SLGBTQIA+ group. The model suggests that for every 1 unit increase on the AMS, there is a 1.34 unit increase in score on the MIA-PSP-emotional sequelae subscale. This finding indicates that anti-mattering may be closely tied to feelings of shame and guilt in sexual and gender minority populations. It is important to consider, however, that the direction of this relationship remains unclear and further investigation is required to determine if anti-mattering precedes MI emotions, or the opposite. The study by Alessi and colleagues [33] highlights the possibility that shame leads to avoidance of social situations. It should be considered that anti-mattering, or feeling invisible and unimportant, may be a factor contributing to an individual’s decision to avoid social encounters or could ultimately be a result of this social avoidance.

### 4.1. Implications

The findings of this study provide novel insights into the stress experienced by individuals who identify as members of gender and sexual minority groups. The variables investigated point to potential sources of risk for negative mental health outcomes among this unique population and identify areas in which we can potentially bolster support at the societal level. This knowledge deepens our understanding of the distress experienced by 2SLGBTQIA+ individuals, which is important given the increased rates of suicidal ideation, PTSD, and depression found among this population [38]. In terms of public health, there is a clear need to promote and actually enact more supportive communities, that is, communities which build an individual’s sense of community mattering [7]. Our results suggest that there is a potential benefit to concentrating efforts towards reducing anti-mattering experienced by these individuals, which could potentially help in mitigating feelings of shame and guilt, along with other forms of distress. These experiences of feeling unlovable, defective, and invisible are underpinned by the core emotional experience of maladaptive shame.

Our findings may have implications for university counselling and support services. Trauma-informed interventions that target students’ felt sense of unimportance may help support student well-being. More broadly, universities may benefit from initiatives that promote supportive campus environments, particularly for students from 2SLGBTQIA+ and other historically marginalized groups. In terms of clinical approaches, integrating components of emotion-focused therapy and self-compassion approaches may afford an antidote to shame by bringing it to light in the presence of a caring other, and in this case, addressing the unmet need of being accepted for who you are [60,61,62]. The ability to hold one’s shame lightly and embrace one’s true self within the context of psychotherapy or with a caring friend or family member can actively change the experience of shame, which thrives when hidden in darkness. The findings speak to the growing need for MI-informed approaches to trauma treatment options [63].

### 4.2. Limitations

As suggested above, the current study relies on correlational self-report data which was gathered from a single university located in a culturally diverse city. Because the study employed a cross-sectional design, causal or directional relationships among the variables cannot be established, and findings should be interpreted as reflecting associations only. It is also important to consider that the use of convenience sampling through an online survey may have led to selection bias, as participation was voluntary with completion more likely by individuals who were sufficiently interested in the study topic. Caution should be used when interpreting the results and generalizing across university students and broader 2SLGBTQIA+ populations. It is likely that experiences will vary greatly between geographical locations and within different subsets of gender and sexual minority communities (e.g., rural versus urban settings, teens versus older adults).

Although the inclusion of 2SLGBTQIA+ participants allowed examination of this underrepresented population, the relatively small sample size and limited representation of gender minority individuals restrict the generalizability of findings and preclude examination of important heterogeneity within the broader 2SLGBTQIA+ community. Additionally, it is important to note that a majority of our 2SLGBTQIA+ sample identified as women and as members of a sexual minority group. Minimal data was obtained from gender minority individuals and male participants, further limiting the ability to generalize our results. While Two-Spirit individuals were included within our broad 2SLGBTQIA+ eligibility criteria, we did not collect sufficient information to identify or analyze Two-Spirit participants as a distinct subgroup. We acknowledge that Two-Spirit identities arise from diverse Indigenous cultural traditions and may reflect sociocultural experiences that differ from those of other sexual and gender minority populations. Consequently, our findings should not be assumed to represent the experiences of Two-Spirit students specifically. Similar studies with larger sample sizes will allow for a more nuanced comparison between subgroups of the 2SLGBTQIA+ sample.

Several limitations surrounding the measures used in our study should also be noted. As mattering was assessed as a global construct, we were unable to examine domain-specific differences among groups (such as peer, family, and university mattering). This may limit interpretation of the findings and should be explored in future research. At the time of study completion, there remained limited tools to assess MI within civilian populations; as such, we were only able to use the MI emotional sequelae subscale from the MIA-PSP. Further work is needed in this area to assess the full construct of MI within the 2SLGBTQIA+ population.

### 4.3. Future Directions

In future studies, it will be valuable to examine mattering and anti-mattering to determine if group differences emerge in specific social groups or settings (e.g., among peers or family). The finding that levels of mattering were similar between groups deserves further investigation, especially considering the differences in anti-mattering that emerged. It may be important to explore if a sense of mattering is strongly present within specific social settings for 2SLGBTQIA+ individuals—such as friend groups or sexual and gender minority communities more broadly—and if the settings that help these individuals to feel that they matter differ from those of individuals who identify as heterosexual and cisgender.

Additional research could investigate MI within sexual and gender minority populations to understand whether perpetrations and betrayals are commonly experienced, and to identify the primary sources of these experiences. New measures may need to be developed to accurately capture the unique experiences of individuals within these groups. With a deeper understanding of the stress experienced by individuals in the 2SLGBTQIA+ community, emphasis can be placed on identifying sources of resilience and fostering support for the mental health of these often-vulnerable individuals.

## 5. Conclusions

The current study established that the experience of distress related to MI is salient among university students who have experienced traumatic events and this distress is associated with levels of anti-mattering. In addition, the results of this study suggested that individuals who identify as members of a sexual or gender minority group encounter heightened childhood adversity and lifetime trauma exposure in comparison to cis gender and heterosexual groups. These individuals were more likely to experience emotional distress associated with MI compared to non-minority individuals, with elevated feelings of shame and guilt. Underpinning such emotional distress, 2SLGBTQIA+ individuals were more likely to perceive that they were insignificant to those around them, which was associated with increased levels of shame and guilt. Interventions aimed at reducing the experience of anti-mattering among trauma-exposed young adults may help to alleviate some of the suffering associated with distressing MI emotions such as shame and guilt.

## Figures and Tables

**Table 1:** Sociodemographic Characteristics of Participants.

	N	%
Gender		
Man	84	22
Woman	287	75
Non-binary, Man/non-binary, Woman/non-binary	8	2
Prefer not to answer/I do not identify with the above	2	1
2SLGBTQIA+		
Yes	52	14
No	316	83
Prefer not to disclose	14	3
Relationship Status		
Married/Cohabiting	35	9
Single	346	91
Racialized Group		
Yes	210	55
No	134	35
Prefer not to disclose	38	10

Note. One participant (*n* = 1) did not report their relationship status, resulting in unequal table sums.

**Table 2:** Pairwise Correlations Among Study Variables for Full Sample.

	ACE	AMS	GMS	MIA-PSP-ES	LEC-5
ACE	-				
AMS	0.34 ***	-			
GMS	−0.13 *	−0.53 ***	-		
MIA-PSP-ES	0.32 ***	0.33 ***	−0.15 **	-	
LEC-5	0.26 ***	0.12 *	0.02	0.19 ***	-

Note. *n* = 382. ACE = Adverse Childhood Experiences Scale; AMS = Anti-Mattering Scale; GMS = General Mattering Scale; MIA-PSP-ES = Moral Injury Assessment for Public Safety Personnel–emotional sequalae subscale; LEC-5 = Life Events Checklist for the DSM-5. *n* = 47 participants did not complete the MIA-PSP-ES. * *p* < 0.05, ** *p* < 0.01, *** *p* < 0.001.

**Table 3:** Pairwise Correlations Among Study Variables for 2SLGBTQIA+ Sample.

	ACE	AMS	GMS	MIA-PSP-ES	LEC-5
ACE	-				
AMS	0.22	-			
GMS	−0.12	−0.55 ***	-		
MIA-PSP-ES	0.21	0.36 **	−0.07	-	
LEC-5	0.21	0.15	−0.02	0.23	-

Note. *n* = 52. ACE = Adverse Childhood Experiences Scale; AMS = Anti-Mattering Scale; GMS = General Mattering Scale; MIA-PSP-ES = Moral Injury Assessment for Public Safety Personnel–emotional sequalae subscale; LEC-5 = Life Events Checklist for the DSM-5. *n* = 2 participants did not complete the MIA-PSP-ES. ** *p* < 0.01, *** *p* < 0.001.

**Table 4:** Pairwise Correlations Among Study Variables for Non-2SLGBTQIA+ Sample.

	ACE	AMS	GMS	MIA-PSP-ES	LEC-5
ACE	-				
AMS	0.32 ***	-			
GMS	−0.12 *	−0.54 ***	-		
MIA-PSP-ES	0.31 ***	0.30 ***	−0.16 **	-	
LEC-5	0.23 ***	0.10	−0.01	0.16 **	-

Note. *n* = 316. ACE = Adverse Childhood Experiences Scale; AMS = Anti-Mattering Scale; GMS = General Mattering Scale; MIA-PSP-ES = Moral Injury Assessment for Public Safety Personnel–emotional sequalae subscale; LEC-5 = Life Events Checklist for the DSM-5. *n* = 45 participants did not complete the MIA-PSP-ES. * *p* < 0.05, ** *p* < 0.01, *** *p* < 0.001.

**Table 5:** Means and Standard Deviations of Survey Measures between the 2SLGBTQIA+ and Non-2SLGBTQIA+ Groups.

Scale	2SLGBTQIA+		Non-2SLGBTQIA+		Significance	Effect Size
	M	SD	M	SD	*p*-Value	Hedge’s g
LEC-5	6.9	3.0	5.6	3.8	0.005	−0.36
MIA-PSP-ES	28.7	10.3	22.2	12.5	<0.001	−0.52
ACE	3.3	2.2	2.0	2.2	<0.001	−0.59
GMS	13.8	3.7	14.2	3.2	0.548	0.10
AMS	14.2	3.6	12.1	4.0	<0.001	−0.53

Note. *n* = 52 for 2SLGBTQIA+ group and *n* = 316 for non-2SLGBTQIA+ group. LEC-5: Life Events Checklist for DSM-5; MIA-PSP-ES: Moral Injury Assessment for Public Safety Personnel–emotional sequalae subscale; ACE: Adverse Childhood Experiences Checklist; GMS: General Mattering Scale; AMS: Anti-Mattering Scale. *n* = 45 participants did not complete the MIA-PSP-ES.

**Table 6:** Regression Results for Variables Predicting MI Emotions in the 2SLGBTQIA+ Group.

	B	S.E.	*p*	*β*
GMS	0.54	0.44	0.225	0.19
AMS	1.34	0.45	0.004	0.47

Note. Adj *R*^2^ = 0.12; GMS = General Mattering Scale; AMS = Anti-mattering Scale.

## Data Availability

The datasets presented in this article are not readily available because the requesting source must be affiliated with an academic institution. Requests to access the datasets should be directed to the corresponding author: jgoldber@yorku.ca.

## Abbreviations

The following abbreviations are used in this manuscript:

MI	Moral Injury
ACEs	Adverse Childhood Experiences
2SLGBTQIA+	Two-Spirit, lesbian, gay, bisexual, transgender, queer, intersex, and asexual communities
